# Research Records as They Stand To-Day

**Published:** 1920-07-31

**Authors:** 


### EXPERIMENTAL STUDIES IN CANCER.

### I.—Research Records as they Stand To-day.

Few medical subjects tend to obtain a greater hold upon the popular imagination than experimental studies on the cause of cancer, but beyond the fact that, up to the present, no satisfactory solution of the problem has been made, there appears to be conspicuously little public realisation of the facts as they really stand. As with most scientific work, the story has been one of slow but steady progress, a gradual accumulation of evidence towards the great discovery which must one day come. And therefore the American *Medical Record* has done wisely in publishing a review under the authorship of M. W. Lyon, M.D., Ph.D., of Indiana, who puts the facts together in a particularly instructive manner.

### The Beginnings of Research.

It was not until the early years of the present century that what might be termed serious experimental work on neoplasms began. Previously little more than a few attempts at transference by direct inoculation had been performed and only after publication of Jensen's work on animal growths in Denmark in 1902, did really painstaking investigation commence. Nowadays at practically all medical centres some work or other is being done upon the cancer problem, and the behaviour of malignant growths in the lower animals is daily becoming better understood. But, unfortunately, this experimental side is essentially confined to manifestations in these lower animals. In man the incidence of tumours is relatively small, occurring neither in epidemics nor yielding an abundance of material that can be studied critically. The inoculation of neoplasms from one person to another, much less a series of persons, cannot be done.

The tumours most frequently studied have been sarcomas (derived from connective tissue) and carcinomas (derived from glandular tissue) occurring spontaneously in the domestic varieties of rats and mice, and artificially propagated in other individuals of the same species. Generally speaking, once a malignant tumour has spontaneously developed, it has been found possible to propagate it almost indefinitely by removing it from the original host and transplanting small portions into many individuals of the same species. There are marked limitations imposed by slight variations in family strain and hereditary character of the animals, particularly in the case of first inoculations; but later, just as with bacteria, the virulence is seen steadily to increase until the percentage of successful inoculations becomes very nearly one hundred. An important point to be noticed is that when, with the tumour portion, a certain amount of connective tissue is also transplanted, the latter is absorbed, and it is in the malignant cells alone that proliferation takes place. On these lines many animal tumours have been kept growing long after the death of the original host. In fact, one of the Jensen mouse cancers is still "under cultivation" after nearly a score of years, although its original host has been dead for many mouse generations.

### Animal and Human Types.

The question naturally arises as to how closely we can compare the animal and human types of growth. From a pathologist's point of view most of the mammalian tumours studied seem to be similar in all essential points to those of man. Also they occur spontaneously in animals at an age comparable with the "cancer age" in man. In experimental work, a difference arises however. In man it is the site of the cancer, and the position of its metastases, or off-shoots, which by their destructive and obstructive functions give rise to a variety of classical signs and symptoms. In the animal, where transplantation is of necessity made in a superficial part of the body, the tumours grow to great sizes before any similar signs of the effects of *internal* neoplasm can occur.

Again, it is impossible to be certain that it is only by true transplantation that transference can occur. Let us quote for example the remarkable chicken-sarcoma of Rous. It upsets many previous conceptions of the pathology of growths. "It was originally found in a Plymouth Rock hen, and carefully transplanted to a series of chickens of the same strain. Its virulence was low at first, but with repeated transfers it became high, yielding 100 per cent. of takes. Subsequent work showed that the juice from this sarcoma after being passed through a moderate-sized filter and entirely cell free, was capable of causing the development of another tumour when inoculated into another fowl. The same is true of the dried and powdered tissue. Its vitality is destroyed by a moderate heating (55° C.) and by antiseptics." The question at once arises, is this a true cancer? Pathologically it is, and yet it appears to be transmitted by a filter-passing virus. It has been transplanted very many times and yet another "original" tumour of the entirely same character has never been found!

### Irritation Tumours.

Another line of investigation has followed the theory of an irritational cause for cancer, and several true irritational tumours have been found in animals. In many cases their origin—call it a "cause" if we will—has been established beyond doubt. And certainly attempts to produce tumours in animals by mechanical means have been partially successful. Records of this part of cancer investigation are singularly interesting in connection with many of the occupational medical problems, and we appear to be on the verge of undoubtedly valuable discoveries.

(*To be continued.*)

